# The Utility of Sodium Channel Provocation in Unexplained Cardiac Arrest Survivors and Electrocardiographic Predictors of Ventricular Fibrillation Recurrence

**DOI:** 10.1161/CIRCEP.122.011263

**Published:** 2022-11-28

**Authors:** Bode Ensam, Christopher C. Cheung, Fahad Almehmadi, Bo Gregers Winkel, Chiara Scrocco, Paul Brennan, Kevin Leong, Alison Muir, Amanda Vanarva, Jacob Tfelt-Hansen, Jason D. Roberts, Andrew D. Krahn, Elijah R. Behr

**Affiliations:** Cardiology Clinical Academic Group, St George’s University Hospitals NHS Foundation Trust, London, United Kingdom (B.E., C.S., E.R.B.).; Institute of Molecular and Clinical Sciences, St George’s University of London, United Kingdom (B.E., C.S., E.R.B.).; Center for Cardiovascular Innovation, Division of Cardiology, University of British Columbia, Vancouver (C.C.C., A.D.K.).; Section of Cardiac Electrophysiology, Division of Cardiology, Department of Medicine, Western University, London, Ontario, Canada (F.A., J.D.R.).; Department of Cardiology, Rigshospitalet, Copenhagen, Denmark (B.G.W., J.T.-H.).; Royal Victoria Hospital, Belfast, United Kingdom (P.B., A.M.).; Imperial College Healthcare NHS Trust, London, United Kingdom (K.L., A.V.).; Section of Forensic Genetics, Department of Forensic Medicine, Copenhagen University, Denmark (J.T.-H.).

**Keywords:** Brugada Syndrome, early repolarization, sodium channel provocation, sudden death, ventricular fibrillation

## Abstract

The implications of a drug-induced type 1 Brugada ECG pattern following sodium channel blocker provocation (SCBP) are not fully understood.

**Methods:**

Baseline clinical and ECG data were obtained from consecutive unexplained cardiac arrest survivors undergoing SCBP at 3 centers. A further 15 SCBP positive (SCBP+) unexplained cardiac arrest survivors were recruited from 3 additional centers to explore ventricular fibrillation recurrence.

**Results:**

A total of 121 consecutive unexplained cardiac arrest survivors underwent SCBP. The yield of the drug-induced type 1 Brugada ECG pattern was 17%. A baseline type 2/3 Brugada pattern (T2/3BP) (adjusted odds ratio, 19.36 [2.74–136.61]; *P*=0.003) and PR interval (odds ratio, 1.03 [1.01–1.05] per ms; *P*=0.017) were independent predictors of SCBP+ response. A pathogenic *SCN5A* variant was identified in 36% of the SCBP+ group versus 0% in the SCBP− group (*P*<0.001). Amongst SCBP+ patients, a spontaneous type 1 Brugada pattern was identified in 19% during follow up and in 24% a type 1 Brugada pattern was identified in a relative. Prior syncope (adjusted hazard ratio, 3.83 [1.36–10.78]; *P*=0.011) and the presence of global early repolarization (hazard ratio, 7.91 [3.22–19.44]; *P*<0.001) were independent predictors of 5-year ventricular fibrillation recurrence. There was a nonsignificant trend toward greater 5-year ventricular fibrillation recurrence in the SCBP− group (23/95 [24%] versus 3/34 [9%]; *P*=0.055).

**Conclusions:**

The yield of the drug-induced type 1 Brugada ECG pattern in consecutive unexplained cardiac arrest survivors undergoing SCBP is 17%. A baseline T2/3BP and PR interval were independent predictors of the drug-induced type 1 Brugada ECG pattern. Greater heritability of BrS phenotype in this group was evidenced by a greater prevalence of pathogenic *SCN5A* variants and relatives with a type 1 Brugada pattern. A history of prior syncope and the presence of global early repolarization were independent predictors of ventricular fibrillation recurrence.

**What is Known?**

- There are concerns regarding the potential for a false positive response to sodium channel provocation with ajmaline.
- Previous studies have indicated a significantly higher yield of the drug-induced type 1 Brugada pattern (DI-T1BP) with ajmaline compared to procainamide across a wide clinical spectrum, but there are no direct head-to-head trials.

**What the Study Adds**

- Sodium channel provocation in a survivor of an unexplained cardiac arrest may uncover a DI-T1BP in 17%.
- In this cohort of patients, there is no statistical difference in DI-T1BP yield between procainamide and ajmaline.
- The 5-year ventricular fibrillation (VF) recurrence rate in those with an unexplained VF arrest was 20% and those with a prior history of syncope or global early repolarization are at the greatest risk, but there is no difference in the incidence of VF recurrence between those with the DI-T1BP and those a negative response to sodium channel provocation.
- In those with the DI-T1BP, the yield a pathogenic *SCN5A* variant is identified in 34% and 27% displayed a spontaneous T1BP during follow up.

Patients surviving an unexpected cardiac arrest due to ventricular fibrillation (VF) are subjected to thorough cardiovascular assessment in order to identify a potential cause. In most cases, coronary angiography or transthoracic echocardiography (TTE) will provide a diagnosis. However, in those without a clear structural, toxicological, or metabolic abnormality, or a baseline electrical disorder, further investigations are often required. In some cases, advanced cardiac imaging with contrast-enhanced cardiac magnetic resonance may identify areas of myocardial fibrosis or motion abnormalities not appreciated on TTE, exercise stress testing, or provocation with epinephrine in those not able to perform exercise stress testing, may unmask long QT syndrome or catecholaminergic polymorphic ventricular tachycardia and sodium channel blocker provocation (SCBP) may identify a concealed form of Brugada syndrome.^1,2^

National registry data suggests the yield of the drug-induced type 1 Brugada pattern (DI-T1BP) following SCBP with procainamide is 6.9% in cardiac arrest survivors with preserved ejection fraction,^3^ while the yield of the DI-T1BP following SCBP with ajmaline has been described in relatives of sudden arrhythmic death syndrome victims, its utility in a large consecutive cohort of cardiac arrest survivors has yet to be reported.^4–7^ Furthermore, the potential for false positives with ajmaline provocation has been highlighted by a Turkish study in which 27.1% of patients with atrio-ventricular nodal re-entrant tachycardia and 4.5% of unrelated controls developed the DI-T1BP following ajmaline provocation.^8^

Following the systematic assessment described above, those patients without a reversible metabolic or toxic cause and not fulfilling the diagnostic criteria for a recognized structural or electrical cardiac disorder may be classified as suffering from idiopathic ventricular fibrillation (IVF). The most recent consensus-derived diagnosis of IVF relies on the exclusion of an alternative structural or primary electrical disorder following comprehensive evaluation.^1^ A meta-analysis of long-term outcomes in patients with IVF suggests the estimated recurrent event rate may be as high at 31% during an average follow-up period of 5 years.^9^

We aimed to determine the yield and significance of the DI-T1BP in a consecutive cohort of survivors with otherwise unexplained cardiac arrest (UCA) undergoing SCBP with either ajmaline or procainamide. We also investigated whether baseline clinical and ECG parameters predict either response to SCBP or the recurrence of VF.

## Methods

### Patient Enrolment

UCA patients were defined as survivors of VF arrest who did not fulfil the diagnostic criteria for a cardiac disorder following: ECG, coronary angiography, cardiac imaging with TTE±cardiac magnetic resonance, and exercise stress testing±epinephrine challenge. Consecutive UCA patients undergoing SCBP were identified at 3 arrhythmia centers: St Georges University Hospital, London, UK; The University of Western Ontario, London, Canada; and the University of British Colombia, Vancouver, Canada. Detailed clinical information and ECGs were shared securely and anonymously with appropriate patient informed consent and local ethical approval was obtained in order to conduct this study. A baseline early repolarization pattern (ERP) was not deemed a contraindication to SCBP and these patients were included in this study. A positive response to SCBP (SCBP+) was defined as the development of the DI-T1BP. The data that support the findings of this study are available from the corresponding author upon reasonable request.

Any future meta-analyses should consider the patients undergoing SCBP with ajmaline as unique to this study; however, a proportion of the patients undergoing SCBP with procainamide have previously been reported as part of earlier registries.^2,3^

### Electrocardiographic Analysis

**Figure 1.**
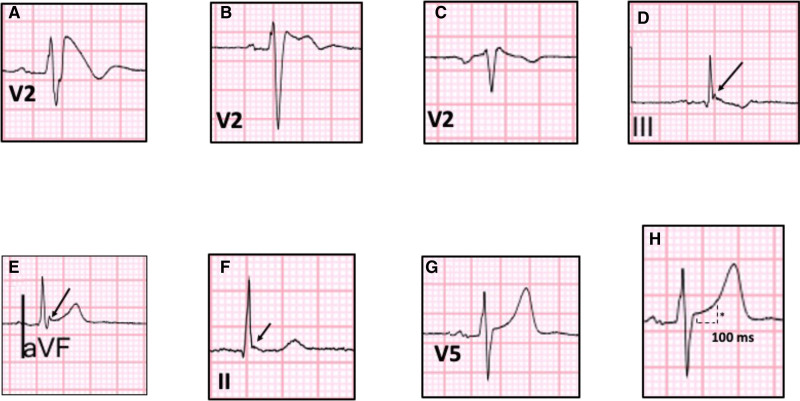
**Electrocardiographic patterns. A**, Type 1 Brugada pattern; **B**, Type 2 Brugada pattern; **C**, Type 3 Brugada pattern; **D**, Terminal QRS notching with downsloping ST segment; **E**, Terminal QRS notching with upsloping QRS ST segment; **F**, Terminal QRS slurring with horizontal ST segment; **G**, Upsloping ST segment; **H**, ST elevation (*) measured at 100 ms from J point.

A resting supine standard 12 lead ECG obtained during the index presentation and prior to the initiation of drug therapy was analyzed for each patient. ECGs acquired in the immediate period following the VF arrest were excluded to avoid the effects of transient metabolic disturbances. A high right precordial lead ECG was used to exclude the presence of a spontaneous T1BP pre-SCBP; however, lead labeling varied between centers and was not used for the 12 lead ECG analysis. Standard electrocardiographic intervals and durations were measured: RR (ms), PR (ms), QT (ms), Bazetts corrected QTc (ms), and QRS (ms). Brugada type 1 and 2/3 patterns were reported according to the standard definitions^10^ (see Figure 1).

An early repolarization pattern, ERP, was defined according to the accepted ECG criteria^11^ as J-point elevation ≥0.1 mV irrespective of concomitant ST segment elevation and included terminal QRS slurring and QRS notching in an anterior, inferolateral, or global ECG distribution. Quantitively, ST segment elevation was measured at 100 ms after the J-point and was subdivided into upsloping and downsloping ST segment, see Figure 1. Mean ST elevation was calculated according to the ECG lead groups as follows: anterior - V1 to V4; lateral - I, aVL, V5, and V6; inferior - II, III, and aVF.

Early repolarization syndrome (ERS) was defined as, an ERP, as described above, in at least 2 contiguous inferolateral leads in an SCBP− patient.^12^

### Recurrent Events

In order to investigate VF recurrence, an additional group of SCBP+ UCA survivors were recruited from 3 arrhythmia centers; Imperial College, London, UK; Royal Victoria Hospital, Belfast, UK; and Copenhagen University Hospital, Copenhagen, Denmark. These UCA survivors underwent an identical investigative pathway as those in the consecutive cohort.

### Statistical Analysis

Data were analyzed with IBM SPSS Statistics version 27. Categorical variables were compared using the χ^2^ or Fisher exact test where appropriate. The Kolmogorov–Smirnov test was used to test the distribution of data. Normally distributed continuous variables were analyzed using a Students *T* test and are reported as mean (±SD) The Mann-Whitney was performed to compare differences between nonnormally distributed continuous variables, which are reported as median [1st quartile to 3rd quartile].

A univariate analysis for the response to SCBP in the consecutive cohort and recurrence of VF in the final overall cohort was performed. Variables demonstrating significant association on univariate analysis (*P*<0.10) were included in a multivariable logistic regression model.

Future event rates are reported as the proportion of the cohort experiencing the primary end point (first implantable cardiac defibrillator [ICD] shock for VF) over a 5-year follow-up period. Those not followed up beyond index presentation were not included in the analysis. A Cox proportional hazards model was used to analyze VF recurrence over a 5-year follow-up period. Hazard ratios (HRs) and 95% CIs are presented. A value of *P*<0.05 was considered statistically significant. Cumulative hazard rates were plotted against time from initial presentation.

## Results

**Figure 2.**
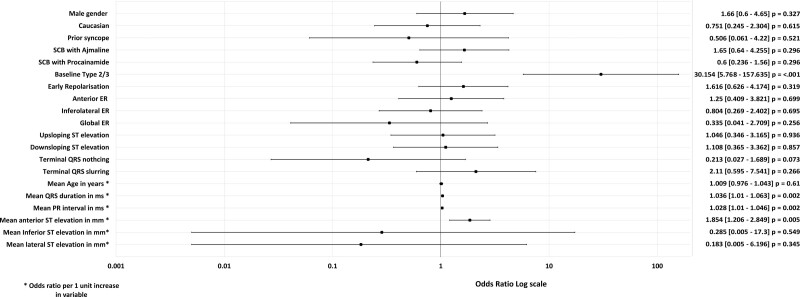
**Univariate analysis for the development of the drug-induced type 1 Brugada pattern (DI-T1BP).** A Forrest plot displaying the odds ratios obtained from the univariate analysis of baseline clinical and electrocardiographic characteristics and sodium channel blockade. A baseline type 2/3 Brugada pattern demonstrated a statistically significant association with the development of the type 1 Brugada pattern, *P*≤0.05. The mean baseline PR interval (ms) and QRS duration (ms) were significantly longer in SCBP+ vs SCBP– patients. ER indicates early repolarization; and SCBP, sodium channel blockade.

**Figure 3.**
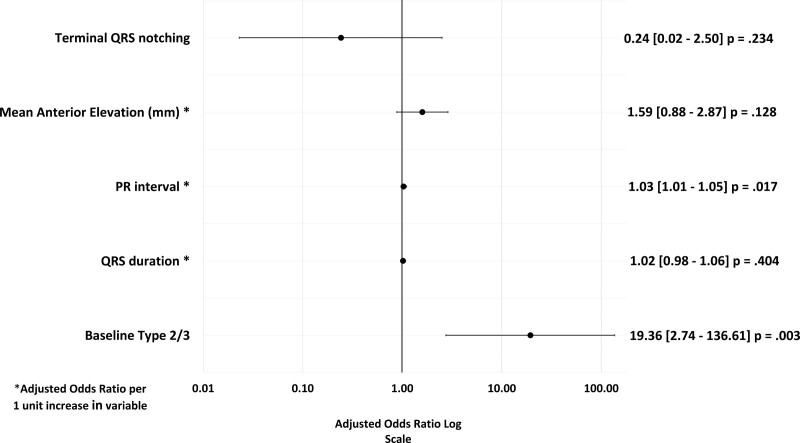
**Multivariable analysis and outcome of sodium channel blockade.** Factors with a *P*≤0.1 in the univariate analysis were included in a multivariable logistic regression model. A baseline type 2/3 Brugada pattern and the mean baseline PR interval (ms) were independently associated with the DI-T1BP. DI-T1BP indicates drug-induced type 1 Brugada pattern.

We identified 121 consecutive UCA survivors undergoing SCBP: the consecutive cohort. An additional 15 SCBP+ UCA survivors from the 3 additional centers were included in the final overall cohort and were included in the analysis of VF recurrence. Table 1 describes the consecutive cohort. Univariate and multivariable regression analyses for factors associated with SCBP+ response are shown in Figures 2 and 3, respectively.

**Table 1.**
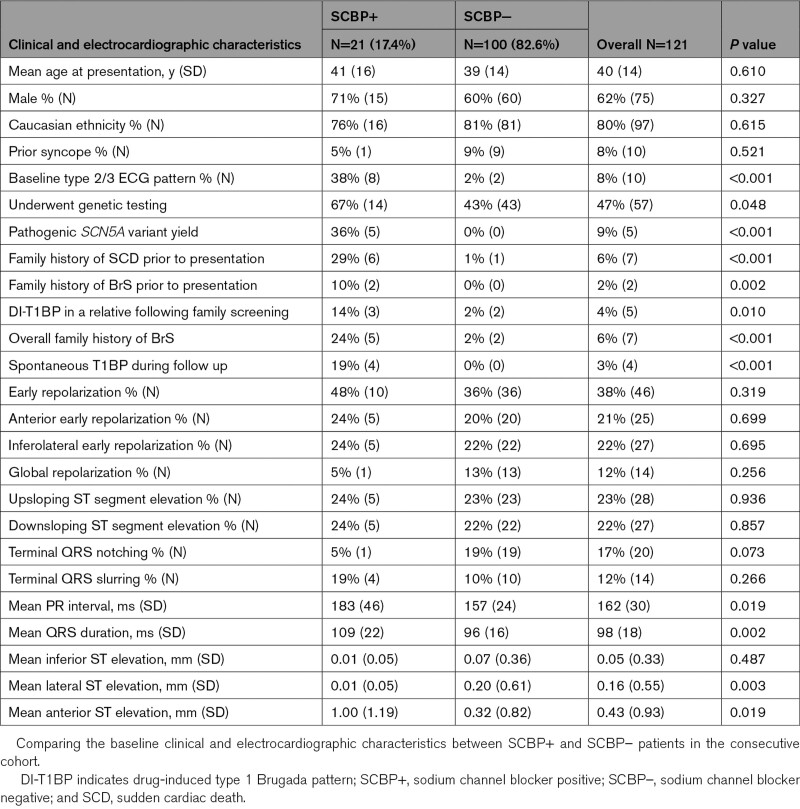
Baseline Characteristics of the Consecutive Cohort

### Yield of the Type 1 Brugada ECG Pattern

In the consecutive cohort, 21 of 121 patients (17.4%) developed the DI-T1BP with SCBP (see Table 1).

The characteristics of SCBP+ versus SCBP− patients showed no significant differences in mean age at index presentation, 41 (16) years versus 39 (14) years (*P*=0.610), respectively; and male proportion, 15 of 21 patients (71%) versus 60 of 100 patients (60%; *P*=0.327).

Across the entire cohort, all patients underwent coronary assessment and a TTE. Two patients were unable to exercise and underwent epinephrine provocation with no finding. A cardiac magnetic resonance was performed in 125 of 136 patients (91.9%) and no major or diagnostic abnormalities were identified. In the 9 patients without cardiac magnetic resonance, the resting TTE was normal.

Of note, 14 of 21 (67%) SCBP+ patients and 43 of 100 (43%) SCBP− patients underwent genetic testing. A pathogenic *SCN5A* variant was identified in 5 of 14 (36%) SCBP+ patients while no pathogenic variants in any gene were identified in any SCBP− patients, *P*<0.001.

There was a family history of sudden cardiac death in a 1st or 2nd degree relative in 6 of 21 (29%) SCBP+ patients versus 1 of 100 (1%) SCBP− patients, *P*<0.001. In addition, prior to presentation, 2 of 21 (10%) SCBP+ patients had a family history of BrS. In contrast, there were no SCBP− patients with a family history of BrS, *P*=0.002. Subsequent familial evaluation revealed a DI-T1BP in a relative in 3 of 21 (14%) SCBP+ patients versus 2 of 100 (2%) SCBP− patients, *P*=0.010. Overall, there was at least one additional family member with a diagnosis of BrS in 5 of 21 (24%) SCBP+ patients versus 2 of 100 (2%) SCBP− patients, *P*<0.001.

Fifty-one patients in the consecutive cohort (42%) underwent provocation using ajmaline, while 70 of 121 (58%) patients were investigated with procainamide. Twenty-two percent (11/51) of those who received ajmaline developed the DI-T1BP compared to 14% (10/70) of patients in patients who underwent provocation with procainamide (*P*=0.211). There were no significant differences in the clinical characteristics of ajmaline-SCBP+ patients and procainamide-SCBP+ patients (Table 2).

**Table 2.**
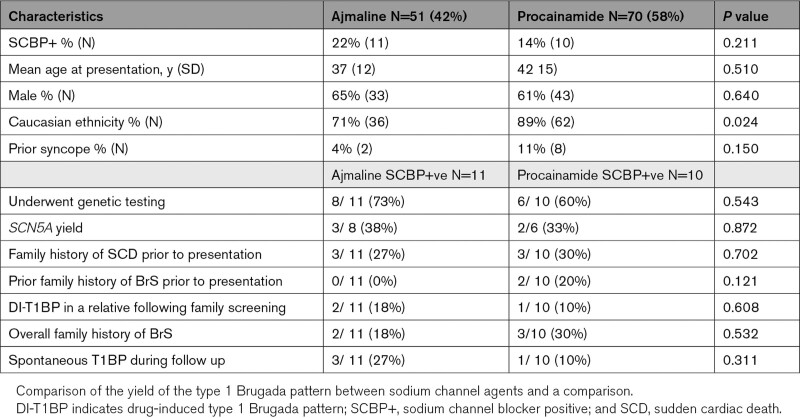
Comparing Sodium Channel Agents

### Electrocardiographic Analysis

The overall prevalence of a baseline type 2/3 Brugada ECG pattern (T2/3BP) across the entire consecutive cohort was 8% (10/121). There was a significantly higher proportion of patients with a baseline T2/3BP within the SCBP+ compared to the SCBP− group (8/21 [38%] versus 2/100 [2%]; *P*<0.001).

The mean baseline PR interval was significantly longer in SCBP+ group of patients compared to SCBP− patients, (183 ms [46 ms] versus 157 ms [24 ms]; *P*=0.019, respectively). The mean QRS duration was also significantly longer in the SCBP+ group (109 ms [22 ms] versus 96 ms [16 ms], *P*=0.002).

An ERP was observed in 46 of 121 patients (38%) of the consecutive cohort. There was no significant difference in its overall prevalence of an ERP between SCBP+ versus SCBP− patients (10/21 [48%] versus 36/100 [36%], respectively; *P*=0.319).

Mean lateral ST segment elevation was significantly greater in SCBP− patients compared to SCBP+ patients (0.20 mm [0.61 mm] versus 0.01 mm [0.05 mm]; *P*=0.003). Conversely mean anterior ST elevation in SCBP+ patients was significantly greater than SCBP− patients (1.00 mm [1.19] versus 0.32 mm [0.82]; *P*=0.019). There was no significant difference in mean inferior ST elevation between SCBP− and SCBP+ patients (0.07 mm [SD=0.36] versus 0.01 mm [SD=0.05]; *P*=0.487; see Table 1).

There was a higher proportion of patients exhibiting inferolateral terminal QRS slurring in the SCBP+ group compared to the SCBP− group, although this was not statistically significant (4/21 [19%] versus 10/100 [10%], respectively; *P*=0.266). Conversely, inferolateral terminal QRS notching was more prevalent in the SCBP− group versus SCBP+ group, but this did not achieve statistical significance (19/100 [19%] versus 1/21 [5%], *P*=0.073).

### Predicting the Response to SCBP

In the univariate analysis, a baseline T2/3BP was a strong predictor of the development of a DI-T1BP (odds ratio [OR], 30.15 [5.77–157.64]; *P*<0.001). A 1 mm (0.1 mV) increase in ST segment elevation in the anterior leads (OR, 1.85 [1.21–2.85]; *P*=0.005), a 1 ms increment in PR interval (OR, 1.03 [1.01–1.05]; *P*=0.002), and 1 ms increment in QRS duration (OR, 1.04 [1.01–1.06]; *P*=0.006) were associated with an increased likelihood of developing the DI-T1BP in the univariate analysis (Figure 2).

In the multivariable logistic regression model (Figure 3), a baseline T2/3BP was an independent predictor of the positive response to SCBP (adjusted OR, 19.36 [2.74–135.61]; *P*=0.003). A 1 ms increase in PR interval continued to be associated with a 3% increasing risk of developing the DI-T1BP (adjusted OR, 1.03 [1.01–1.05]; *P*=0.017).

### VF Recurrence

The characteristics of the additional cohort of 15 SCBP+ UCA patients are described in the Supplemental Material. The ICD implant rate across the final cohort of 136 patients was 100%. Follow-up data were available for 129 of 136 (95%) patients with a median follow-up period of 6.20 years (3.24–9.75 years).

Over a 5-year follow-up period, 26 of 129 patients (20%) experienced an appropriate ICD shock for VF. The mean time to recurrence of VF was 2.13 years (1.37 years). There was a trend toward an increased risk of VF recurrence over the 5-year follow-up period in the SCBP− group (23/95 [24%] versus 3/34 [9%]); however, this did not reach statistical significance (HR, 2.54 [0.76–8.47] versus 0.31, [0.04–2.28]; *P*=0.128).

An analysis of the ICD electrocardiograms of those experiencing VF during a 5-year follow-up period identified short-coupled premature ventricular contractions (coupling interval <350 ms) triggering VF storm in 67% (2/3) of SCBP+ patients and 52% (12/23) of SCBP− patients.

### Predicting VF Recurrence

**Figure 4.**
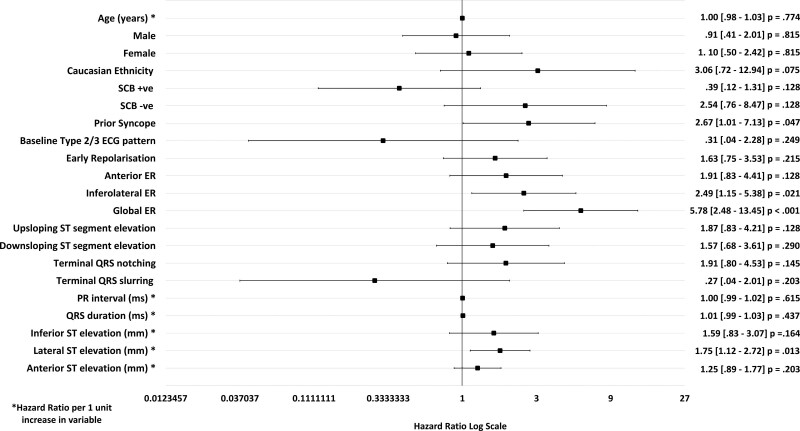
**Baseline clinical and electrocardiographic factors predicting the 5-year recurrence of ventricular fibrillation.** A Forrest plot displaying the hazard ratios obtained from the univariate analysis of a clinical and electrocardiographic characteristics and recurrence of ventricular fibrillation (VF) over a 5-year follow-up period. Prior syncope, early repolarization (ER) in a global or inferolateral distribution and degree of ST segment elevation in the lateral leads were associated with an increased risk VF recurrence. SCBP indicates sodium channel blockade.

**Figure 5.**
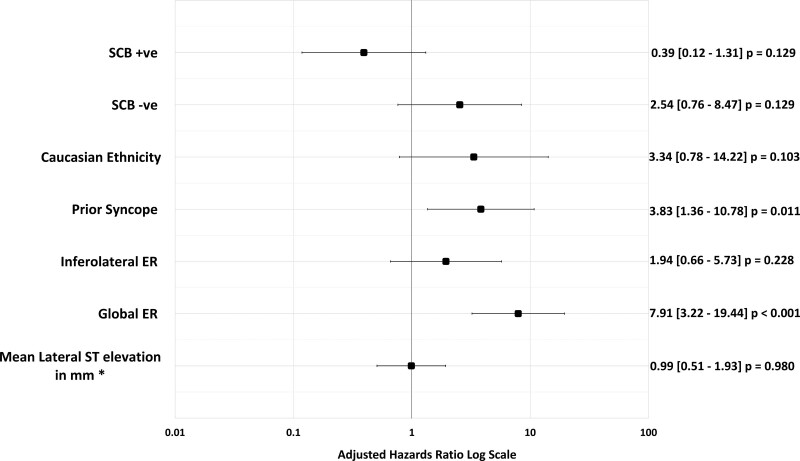
**Multivariable analysis and 5-year recurrence of ventricular fibrillation.** Factors with a *P*≤0.1 in the univariate analysis were included in a multivariable logistic regression model with sodium channel outcome and Caucasian ethnicity as co-variates. Prior syncope and global early repolarization (ER) were independent predictors of ventricular fibrillation recurrence over a 5-year follow-up period. SCBP indicates sodium channel blockade.

A history of prior syncope (HR, 2.67 [1.01–7.13]; *P*=0.047), an ERP in an inferolateral distribution (HR, 2.49 [1.15–5.38]; *P*=0.021) and global distribution (HR, 5.78 [2.48–13.45]; *P*<0.001), along with increasing lateral ST elevation (HR, 1.75 [1.12–2.72]; *P*=0.13) were predictors of a recurrence of VF in the univariate analysis (Figure 4). In the multivariable model, prior syncope (HR, 3.83 [1.36–10.78]; *P*=0.01) and the presence of a global ERP (HR 7.78, [3.18–19.02]; *P*<0.001) were independent predictors of VF recurrence over a 5-year period (Figure 5).

**Figure 6.**
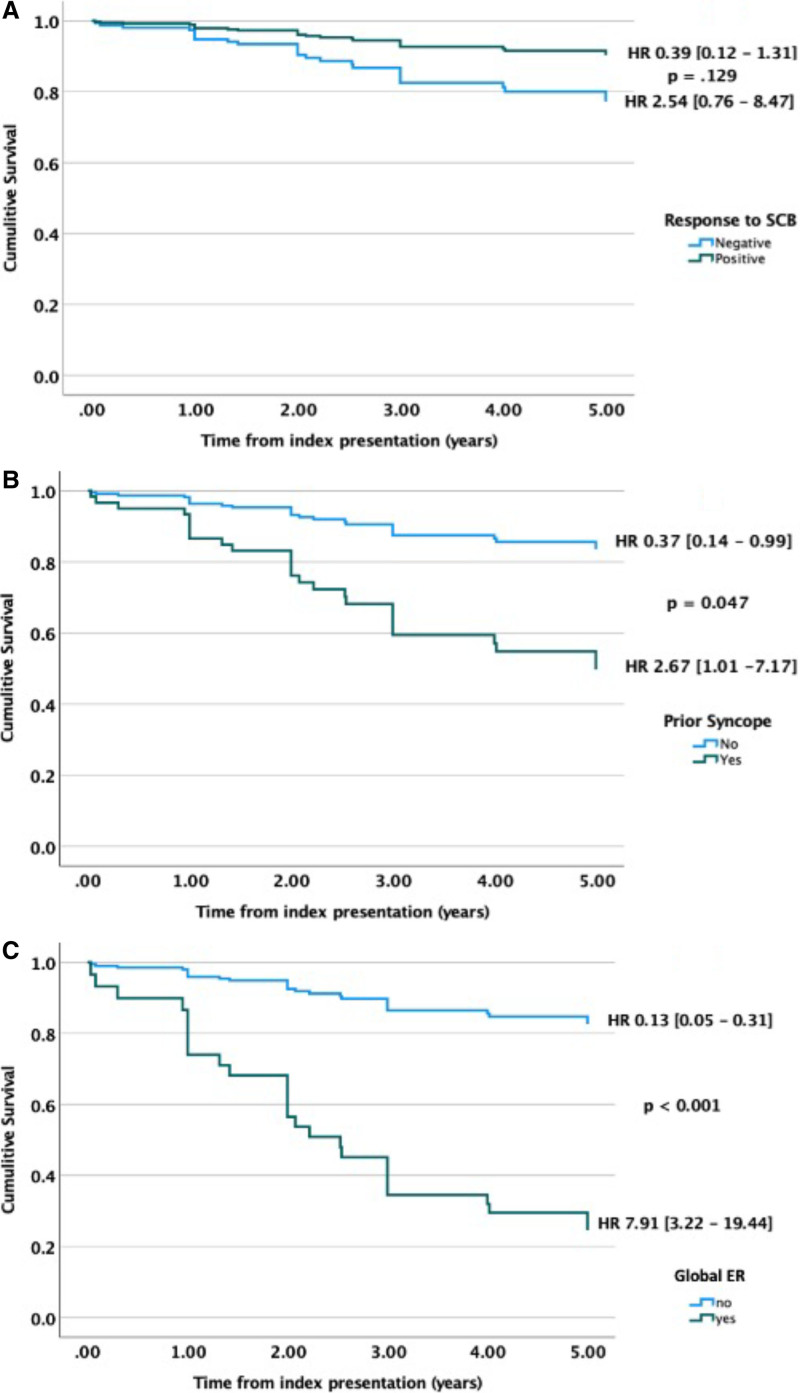
**Cumulative survival plots.** Survival plots comparing 5-year recurrence of ventricular fibrillation against time from index presentation for (**A**) sodium channel blocker outcome, (**B**) prior syncope, (**C**) global early repolarization. ER indicates early repolarization; HR, hazard ratio; and SCB, sodium channel blockade.

Cumulative 5-year survival analysis for SCBP outcome, prior syncope, and global ER are shown in Figure 6.

## Discussion

In this multicenter retrospective study, we identified 121 consecutive patients undergoing SCBP after UCA. The overall yield of the DI-T1BP was 17.3% with no significant difference between ajmaline (22%) and procainamide (14%). The presence of a baseline T2/3BP was a strong and independent predictor of SCBP+ response (DI-T1BP), as was PR interval (ms). Across the entire final cohort, 20% experienced a recurrence of VF over a 5-year follow-up period with a trend toward an increased risk of recurrence in the SCBP− group. A history of prior syncope and the presence of an ERP in a global distribution on the baseline 12-lead ECG were independent predictors of VF recurrence.

### False Positive or True Positive?

Currently, a DI-T1BP and a history of aborted cardiac arrest are sufficient to make a diagnosis of BrS and thus 21 of 121 patients (17%) of our cohort fulfilled this criterion.^13,14^ However, considering the reported potential for false positive outcomes with SCBP,^8^ especially with ajmaline, we sought to provide additional evidence in support of a true positive result and diagnosis of BrS in those developing the DI-T1BP.

First, 36% of the SCBP+ patients in our study were found to have pathogenic *SCN5A* variants following clinical genetic testing. This is higher than the overall reported yield of 20% to 25%. The utility of genetic testing in those without a diagnosis following an UCA is limited, as such only 43% of our SCBP− cohort underwent clinical genetic panel testing, with no pathogenic variants being identified.

In accordance with the current guidance, screening with SCBP in family members of our SCBP+ patients was undertaken consistently, and we observed a DI-T1BP in at least one relative in 14% of this group. In contrast, the extent of phenotypic evaluation in the SCBP− cohort varied and while we observed the DI-T1BP in only 2 relatives of separate unrelated SCBP− patients, the yield may have been higher if SCBP had been systematically applied in this group. These 2 cases have previously been described in a study by Mellor et al.^15^ In families 2 and 3 of this study, a relative of the UCA survivor was found to have a type 2 BrS on subsequent family screening, which converted to a T1BP with ajmaline. In both cases, SCBP in the UCA survivor was repeated and remained negative. Overall, Mellor et al identified a DI-T1BP in at least one family member in 12 UCA probands giving an overall positive rate of 25% (3/12) at the family level. Whether this represents a false positive DI-T1BP in the relative or a false negative in the UCA survivor is difficult to determine. This outcome may be affected by several factors, including the age of the UCA victims, all <21 years of age. Variable expressivity mediated by common genetic variation or environmental factors might influence the outcome of SCBP in these subjects.^16^

While we also observed that 10% (2/21) of SCBP+ patients had a family history of BrS prior to presenting with an UCA, neither of these subjects had previously been screened for BrS. There was no prior history of BrS in the SCBP− group.

Overall, 19% (4/21) of the SCBP+ cohort were found to have a spontaneous T1BP observed on a resting ECG during follow up, as opposed to ambulatory ECGs. Gray et al^17^ reported a burden of the spontaneous T1BP in patients with a DI-T1BP of 34% (11/32). However, the utility of a high lead ambulatory ECG recording in a patient already receiving an ICD is uncertain and was thus not performed routinely in our study. The presence of a spontaneous T1BP, however, strengthens the diagnosis of BrS in a fifth of our SCBP+ cohort.

Taken together these data support an enrichment of the DI-T1BP group for BrS as a diagnosis, although in the absence of a gold standard, the possibility of false positives and false negatives remains.

### Ajmaline Versus Procainamide

While there is no direct head-to-head or cross over trial data, previous studies have reported a consistently higher yield of the DI-T1BP with ajmaline in comparison with procainamide across clinical indications. Papadakis et al^4^ observed the ajmaline DI-T1BP in 15.9% of patients undergoing SCBP following a diagnosis of sudden arrhythmic death syndrome in a relative. Similarly, Tadros et al^6^ reported a yield of 20.4% in UCA probands and 14% in family members of sudden arrhythmic death syndrome/UCA victims. Somani et al^3^ reported a yield of the DI-1TBP in 6.9% in a mixed cohort undergoing SCBP with procainamide.

In this current study, the yield of the DI-T1BP observed in the group receiving procainamide was higher than previously reported with this SCBP agent in similar cohorts, and while the trend to a higher yield with ajmaline continued, this was not statistically significant. This may suggest that the yields of the 2 agents are comparable when applied to those deemed to carry the greatest a priori chance of having a more penetrant form of BrS, that is, presenting with an otherwise unexplained cardiac arrest.

There were no other differences in the clinical characteristics in ajmaline-SCBP+ and procainamide-SCBP+ patients, with similar yields following genetic testing and familial evaluation. There was a trend toward a greater proportion of procainamide SCBP+ patients having a family history of BrS, but this was not significant. A spontaneous T1BP was seen during follow-up in an ajmaline-SCBP+ patient more frequently than in a procainamide-SCBP+ patient, but this was not statistically significant.

#### SCBP Positive Versus SCBP Negative: Electrocardiographic Comparisons

SCBP+ patients, as expected, displayed a higher prevalence of the T2/3BP at baseline, a greater mean ST elevation (mm) in the anterior leads and longer mean PR (ms) intervals and mean QRS (ms) durations, while SCBP negative patients demonstrated a greater mean lateral ST elevation (mm). Baseline T2/3BP and an increasing PR interval, a novel finding, were independent predictors of the positive response to SCBP.

Prolongation of the QRS duration and PR interval have previously been reported in patients with BrS and are both associated with an increased incidence of major arrhythmic events (syncope, ventricular tachycardia, VF, and appropriate ICD shocks).^18–22^ The association between major arrhythmic events and QRS duration and PR interval prolongation in BrS patients in general appears to be irrespective of genetic status; however, a relationship between *SCN5A* pathogenic variants and cardiac conduction disease in BrS is well described.^18,23^ However, all patients in our series had already suffered a cardiac arrest. These ECG parameters, therefore, appear less relevant as risk makers for recurrence of VF.

Five-year VF recurrence rates, time to recurrence, and estimated 5-year cumulative survival rates were statistically comparable between the 2 groups, although there was trend toward greater risk of 5-year VF recurrence rates in the SCBP− group (HR, 2.54 [0.76–8.47] versus HR, 0.39 [0.12–1.31]; *P*=0.129).

#### Early Repolarization

The first description of ERS relied on the presence of elevation of the QRS-ST segment ≥1 mV in at least 2 contiguous leads in patients with UCA.^24^ Subsequent electrocardiographic refinement included an assessment of the ST segment slope, upsloping or downsloping, with the latter having a greater arrhythmic risk especially if located in the inferior and or lateral leads in the general population^25^ and a greater risk of VF recurrence in UCA survivors. The current consensus definition accepts that an ERP may exist in the absence of ST segment elevation if there is J point elevation >0.1 mV either as a notch or as slurring within the terminal QRS^11^ (central illustration). In our study, 22% of the SCBP− group had inferolateral ER >2 contiguous leads on the baseline ECG at presentation and would therefore fulfil the current consensus definition of ERS.^13^ However, a similar proportion of the SCBP+ group also displayed baseline inferolateral ER, 24%, *P*=0.695.

Antzelevitch et al^26,27^ have previously sought to classify ER into subtypes based on spatial distribution and increasing arrhythmic risk with type 1 being benign ER isolated to the lateral precordial leads, type 2 associated with a greater risk and present in the inferolateral leads, type 3 showing a global distribution, and type 4 being J-point elevation related to the DI-T1BP.

We explored the influence of the individual components of ER. Mean lateral ST segment elevation was significantly greater in the SCBP− group in contrast to a greater degree of anterior ST elevation in the SCBP+ group. The pattern of distribution of ST segment elevation was comparable between the groups; however, the SCBP− group demonstrated a trend toward a greater prevalence of global ST segment elevation, which was the only electrocardiographic feature with a significant independent association with VF recurrence. This is consistent with type 3 ERS described by Antzelevitch et al. However, the HRs for VF recurrence were comparable between the upsloping and downsloping subtypes of ST elevation. This differs from prior work by Rosso et al,^28^ although the comparator group was an athletic control and the patient group was smaller than that presented here.

#### Syncope

While the overall prevalence of prior syncope across the entire population was low (8%), it did prove to be an independent predictor of VF recurrence across our population (adjusted HR, 3.83 [1.36–10.78], *P*=0.011). The prevalence of syncope pre-enrolment in BrS registries has been reported to be between 21% and 34%.^29–32^ However, much of these data are from those without prior cardiac arrest. Nevertheless, in those with syncope, a significantly higher incidence of VF or appropriate ICD therapy was observed.

The Cardiac Arrest Survivors With Preserved Ejection Fraction Registry reported a higher prevalence of prearrest syncope than that observed in our study; 35% across the entire cohort and 32% in those in whom the final diagnosis was IVF and 37% in those with an alternative diagnosis.^2^ However, a recent subgroup analysis by Steinberg et al^33^ noted an absence of prearrest syncope in IVF patients with VF recurrence triggered by short-coupled premature ventricular contractions (<350 ms coupling interval). The authors excluded those with a diagnosis of ERS. Our cohort may reflect an intermediate or mixed phenotype as we did not exclude SCA survivors with triggering short-coupled premature ventricular contractions.

#### Clinical and Mechanistic Implications

The development of a DI-T1BP in UCA survivors does not appear to be associated with an increased risk of further VF and one could argue that the utility of SCBP in this setting is therefore limited. However, with 14% of SCBP+ patients having at least one relative with a DI-T1BP on subsequent cascade screening, as well as the greater prevalence of a FH of sudden cardiac death and BrS and pathogenic genetic variants, SCBP remains an important tool in the identification or exclusion of a heritable trait.

Previous work by Nademanee et al,^34^ in a cohort of patients with recurrent VF, ER ± co-existing Brugada ECG patterns, identified the presence of epicardial low voltage and fractionated late potentials in the RVOT and inferolateral RV in those exhibiting both patterns. The timing of these late potentials corresponded to the presence of J point elevation on the surface ECG (predominantly in the inferolateral leads) and subsequent substrate ablation resulted in a drastic reduction in VF recurrence. We observed a similar inferolateral distribution of J-point ST segment elevation in 28% of our final cohort, which was associated with an increased risk of VF recurrence within the 5-year follow-up period in the univariate analysis (HR, 2.49 [1.15–5.38]; *P*=0.021).

Histopathologic assessment of biopsied tissue from a patient with ERS identified extensive myocardial fibrosis, which corresponded with fractionated potentials, delayed activation, and repolarization abnormalities during epicardial mapping of the inferior right ventricular free wall, which were associated with an ER pattern on the surface electrocardiogram.^35^ Ablation of these regions resulted in a reduction in arrhythmia recurrence and normalization of the ECG. Furthermore, genetic studies have shown that *SCN5A* pathogenic variants are important in ERS as well as BrS patients. Zhang et al^36^ reported a 10% yield of likely pathogenic and pathogenic *SCN5A* variants in a cohort of ERS probands compared with a yield of 23% in BrS probands. Similarities in the clinical and demographic characteristics between these groups were noted but electrocardiographic differences with the BrS *SCN5A* positive probands demonstrating significantly longer QRS durations, shorter PR intervals, longer QTc intervals, and a lower prevalence of bradycardia compared to the ERS *SCN5A* positive group. Interestingly, the authors also described a patient with a fever-induced ERS phenotype with a *SCN5A* pathogenic variant and fever-induced BrS phenotype in another patient with the same variant.

The evidence suggests that ERS and BrS may in part share common histopathological substrates and mechanisms for arrhythmogenesis, which are manifested by J-wave changes on the surface ECG. Our study suggests that the location and extent of these J waves, some of which may be augmented by SCBP challenge and some not, appears to determine the risk of recurrence of VF. We, therefore, need to change our approach to the categorization and management of UCA patients as syndromes, and better understand the underlying epicardial substrate of the J wave syndrome. In this way, we will be able to manage patients for their individual risk for VF and recurrence and offer catheter ablation of substrates in an effective way.

### Study Limitations

The centers participating in this study are either national or regional referral centers for inherited arrhythmia syndromes. There may be patients referred with an UCA in whom we were able to find a cause without the need for SCBP. These patients were not included in this study. Additionally, it would be difficult to determine whether there were eligible UCA patients who were not referred. As such the true number of patients presenting with an UCA is unknown and we were unable to provide any prevalence data.

While statistical power may be a potential reason for the lack of statistically significant associations for certain analysis. The number of patients in the SCBP+ and VF recurrence groups were small and represented <20% of the total cohort. This may overestimate the strength of the effect in the multivariable analysis; however, there are few comparable studies of this size investigating similar cohorts or phenotypes in this manner.

While our cohort of patients were thoroughly investigated at the point of presentation, there is evidence to support the late development of structural disorders in patients given an initial diagnosis of IVF.^37^ We report on recurrent event rates our cohort, but data regarding the evolution of structural disorders were not available.

### Conclusions

This study has identified a 17% of the DI-T1BP in consecutive UCA survivors undergoing SCBP, with no significant difference observed between SCBP agents. The presence of a baseline T2/3BP was an independent predictor of a DI-T1BP as was an increasing PR interval suggesting a greater degree of conduction abnormality in this group. Prior syncope and global ER were independent predictors of VF recurrence. A DI-T1BP response did not, however, show an independent association with recurrence but may be a marker of a more heritable form of the condition. This may be consistent with the accumulating view that many IVF survivors share a similar epicardial fibrotic substrate, the extent and location of which may mediate their risk for VF recurrence. This needs to be studied to enable accurate risk evaluation in less severely affected individuals with BrS and ERS and their family members.

## Article Information

### Sources of Funding

This work is supported by The Robert Lancaster Memorial Fund sponsored by McColl’s Research Group (B. Ensam, C. Scrocco, and E. Behr) and the Heart and Stroke Foundation of Canada (A. Krahn).

### Disclosures

None.

### Supplemental Material

Table S1

## Supplementary Material


