# Supplementary material for: The Utility of Sodium Channel Provocation in Unexplained Cardiac Arrest Survivors and Electrocardiographic Predictors of Ventricular Fibrillation Recurrence
Source: Circ Arrhythm Electrophysiol. 2022 Nov 28;15(12):e011263. doi: 10.1161/CIRCEP.122.011263 (PMC10289235; doi:10.1161/CIRCEP.122.011263)
Supplement: Supplementary file 1 [file hae-15-e011263-s001.pdf]

SUPPLEMENTAL MATERIAL

Supplementary Table I

**Characteristics**	**Selected SCBP+ patients**
	**N = 15**
**Mean age at presentation (years) (SD)**	39 (12)
**Male % (N)**	93 (14)
**Caucasian Ethnicity % (N)**	73% (11)
**Prior Syncope % (N)**	7% (1)
**Baseline Type 2/3 ECG pattern % (N)**	33% (5)
**Early Repolarisation % (N)**	53% (8)
**Anterior Early Repolarisation % (N)**	40% (6)
**Inferolateral Early Repolarisation % (N)**	33% (5)
**Global Early Repolarisation % (N)**	7% (1)
**Upsloping ST segment elevation % (N)**	47% (7)
**Downsloping ST segment elevation % (N)**	13% (2)
**Terminal QRS notching % (N)**	13% (2)
**Terminal QRS slurring % (N)**	20% (3)
**Mean PR interval (ms) (SD)**	175 (30)
**Mean QRS duration (ms) (SD)**	95 (15)
**Mean Inferior ST elevation (mm) (SD)**	.13 (.51)
**Mean Lateral ST elevation (mm) (SD)**	.26 (.59)
**Mean Anterior ST elevation (mm) (SD)**	.80 (.77)

Clinical and electrocardiographic characteristics of the selected unexplained cardiac arrest sodium channel blocker positive patients
